# DCD-VMUNet: A Dynamic Multi-Scale and Geometry-Aware Network for Coronary Vessel and Stenosis Segmentation in X-Ray Angiography

**DOI:** 10.3390/bioengineering13101111

**Published:** 2026-09-24

**Authors:** Youzhuo Quan, Huaizhi Yue, Xiangyang Ma, Kun Liu

**Affiliations:** 1 Department of Cardiology, Union Hospital, Tongji Medical College, Huazhong University of Science and Technology, Wuhan 430022, China; 2 Hubei Key Laboratory of Biological Targeted Therapy, Union Hospital, Tongji Medical College, Huazhong University of Science and Technology, Wuhan 430022, China; 3 Hubei Engineering Research Center for Immunological Diagnosis and Therapy of Cardiovascular Diseases, Union Hospital, Tongji Medical College, Huazhong University of Science and Technology, Wuhan 430022, China; 4 School of Information Engineering, Chang’an University, Xi’an 710064, China

**Keywords:** X-ray coronary angiography, coronary vessel segmentation, coronary stenosis localization, medical image segmentation, deep learning

## Abstract

Accurate segmentation of coronary vessels and stenotic regions in X-ray coronary angiography remains challenging because of low vessel-to-background contrast, vessel overlap, thin branches, and the small, ambiguous appearance of stenotic lesions. In this study, we propose DCD-VMUNet, a structure-aware framework for automated coronary vessel and stenosis segmentation. Built on VM-UNet, the model incorporates a dynamic multi-scale kernel module, a centerline head, and a direction head. The dynamic module adaptively integrates fine boundary details with broader contextual cues, while the auxiliary heads introduce geometric supervision to enhance structural continuity and orientation awareness. DCD-VMUNet was independently trained and evaluated for binary vessel and stenosis segmentation on the ARCADE dataset. It was compared with YOLOv11-X, DeepLab v3+, UNet, UNet++, TransUNet, MALUNet, and VM-UNet using pixel-wise metrics and, for vessel segmentation, the centerline-aware clDice metric. For vessel segmentation, DCD-VMUNet achieved the best performance, with IoU, precision, recall, and F1-score of 0.6838, 0.8418, 0.7846, and 0.8122. For stenosis segmentation, it also ranked first across all metrics, reaching 0.4042, 0.5826, 0.5688, and 0.5757, respectively. Ablation studies confirmed the complementary effects of multi-scale feature extraction and geometric supervision. Additional evaluation on the independent ICA dataset further supported the vessel segmentation performance of DCD-VMUNet beyond the ARCADE benchmark. These results indicate that DCD-VMUNet improves coronary vessel delineation and pixel-level stenosis localization, supporting automated coronary angiographic assessment.

## 1. Introduction

Cardiovascular disease remains a major contributor to global mortality and disability, with ischemic heart disease representing one of its principal components [1]. In patients with coronary artery disease (CAD), atherosclerotic plaque progression and plaque-related thrombosis can narrow or obstruct the coronary lumen, thereby compromising myocardial blood supply [2]. Reliable characterization of coronary anatomy and stenosis severity is therefore clinically important, particularly when treatment planning involves potential coronary revascularization. Contemporary revascularization guidelines also emphasize that treatment decisions should consider coronary disease complexity, technical feasibility, and multidisciplinary clinical judgment [3]. Thus, quantitative assessment of coronary stenosis provides essential anatomical information for clinical assessment and therapeutic planning.

Invasive coronary angiography remains the reference standard for anatomical assessment of coronary stenosis and plays a central role in CAD management [4]. However, routine stenosis grading still relies largely on physicians’ visual interpretation, which can differ substantially from quantitative coronary angiography and varies across physicians and hospitals [5]. From an image-analysis perspective, automated coronary angiographic analysis remains challenging because of projection-related vessel overlap, motion artifacts, low vessel-to-background contrast, and interference from nonvascular structures [6,7,8]. These limitations motivate the development of automated and reproducible methods for coronary artery segmentation and stenosis localization.

Artificial intelligence (AI), particularly machine learning and deep learning, has been increasingly applied in medicine, with applications ranging from disease diagnosis and risk assessment to automated medical image analysis and segmentation [9]. Automated coronary artery segmentation is a key step toward reproducible computer-assisted analysis of X-ray coronary angiography. U-shaped encoder–decoder networks, represented by U-Net, have become a widely used architecture for biomedical image segmentation because they combine contextual feature extraction with spatially precise localization [10]. In coronary angiography, deep learning methods have shown promising performance for major-vessel segmentation. Yang et al. reported that fully convolutional deep learning models could accurately segment major coronary vessels in X-ray coronary angiograms [11], while AngioNet further demonstrated the feasibility of automatic vessel segmentation for supporting systematic anatomical assessment of coronary stenosis [12]. More recently, Transformer-based and state-space-based architectures have been introduced into medical image segmentation to enhance global contextual modeling beyond the local receptive field of conventional convolutional operations [13,14]. These advances provide important methodological foundations for automated coronary angiographic analysis. Existing medical image segmentation methods encompass conventional convolutional architectures, U-shaped encoder–decoder networks, transformer-based models, and more recent state-space-model-based approaches, which provide different capabilities for local feature extraction, spatial localization, global context modeling, and efficient long-range dependency modeling [10,13,14]. Compared with these existing segmentation approaches, DCD-VMUNet further incorporates dynamic multi-scale feature extraction together with centerline- and direction-aware auxiliary supervision to better preserve vascular structure and orientation information.

Despite this progress, segmentation of coronary vessels and stenotic lesions remains technically challenging. At the segmentation level, the imaging limitations of X-ray coronary angiography are further compounded by the thin, elongated, and branching morphology of coronary arteries. Accurate vessel delineation therefore depends not only on pixel-wise overlap but also on preservation of vascular continuity and topology. This issue is particularly relevant for tubular structures, for which centerline-based topological consistency has been shown to provide information that conventional region-overlap metrics may not fully capture [15]. Stenosis segmentation is even more demanding because stenotic lesions are localized abnormalities within the vessel tree and may occupy only small, visually ambiguous regions. The ARCADE benchmark explicitly separates coronary vessel analysis from stenosis detection by providing expert annotations for coronary artery segments and stenotic plaque locations, highlighting the need for models that can address both anatomical vessel structures and pathological regions [16].

In this study, we propose DCD-VMUNet, a structure-aware segmentation network for coronary angiographic images. The proposed model is built upon VM-UNet, which adopts visual state-space modeling to capture long-range contextual information with linear computational complexity [14]. To better adapt this framework to coronary angiography, DCD-VMUNet incorporates a dynamic multi-scale kernel module, a centerline head, and a direction head. The dynamic multi-scale kernel module is designed to enhance the representation of vessels and stenotic lesions with varying calibers, shapes, and spatial extents, drawing on the general principle that adaptive receptive-field selection can improve multi-scale feature representation [17]. The centerline head introduces auxiliary structural supervision to strengthen vessel continuity, whereas the direction head guides the network to learn the orientation characteristics of elongated and tortuous coronary vessels. By integrating appearance, topology, and directional cues, DCD-VMUNet aims to improve the segmentation of both coronary vessels and stenotic lesions.

The main contributions of this work are threefold. First, we propose DCD-VMUNet for pixel-level coronary vessel and stenotic-region segmentation in X-ray coronary angiography. Second, we introduce dynamic multi-scale feature extraction together with centerline- and direction-aware auxiliary supervision to enhance vascular continuity, local boundary representation, and directional consistency. Third, we comprehensively evaluate DCD-VMUNet on the ARCADE benchmark using pixel-wise and centerline-aware metrics together with statistical and reproducibility analyses, and further validate its vessel segmentation performance on the independent ICA dataset.

## 2. Method

In this section, we describe the proposed DCD-VMUNet framework for coronary angiographic image segmentation. The same architecture was independently trained and evaluated for two binary segmentation tasks: coronary vessel segmentation and stenosis segmentation. We first introduce the ARCADE dataset and the preprocessing strategy used in this study, and then present the overall architecture of DCD-VMUNet.

DCD-VMUNet is developed based on the VM-UNet framework and enhanced by three components: the dynamic multi-scale kernel (DMSK) module, the centerline head, and the direction head. The DMSK module strengthens multi-scale feature representation, while the centerline and direction heads introduce auxiliary geometric supervision to improve structural continuity and orientation awareness. These components are jointly integrated to improve the segmentation accuracy and robustness of coronary vessels and stenotic regions. Figure 1 illustrates the overall workflow of the proposed method.

### 2.1. ARCADE and ICA Datasets

Two publicly available X-ray coronary angiography (XCA) datasets were used in this study: the ARCADE dataset for the primary evaluation of coronary vessel and stenosis segmentation, and the ICA dataset for additional validation of coronary vessel segmentation.

The Automatic Region-based Coronary Artery Disease Diagnostics using X-ray Angiography Images (ARCADE) dataset [16] was used to evaluate the proposed DCD-VMUNet for coronary vessel and stenosis segmentation. ARCADE is a publicly available X-ray coronary angiography dataset designed for automated coronary artery disease analysis and contains two segmentation-related tasks: coronary artery segmentation and stenosis localization.

For both coronary artery and stenosis segmentation, the dataset provides 1500 XCA images, including 1000 training images, 200 validation images and 300 test images. The annotations follow a SYNTAX Score-based protocol, in which coronary arteries are labeled according to anatomically defined vessel segments. In this study, all annotated coronary artery segments were merged into a single foreground class to generate binary vessel masks. For stenosis segmentation, the corresponding stenotic regions were also converted into binary lesion masks, with lesion pixels treated as foreground and all remaining pixels treated as background. For each ARCADE task, the official training, validation and test split provided by the dataset was used. The validation set was used for model selection and selection of the best-performing checkpoint, whereas the test set was used exclusively for the final performance evaluation and was not involved in model selection.

To further evaluate the effectiveness of the proposed method on an independent dataset, we additionally employed the publicly available ICA dataset [18] for coronary vessel segmentation. The dataset consists of XCA images with corresponding pixel-level binary vessel masks and has been used in previous studies for coronary vessel segmentation. Since the ICA dataset provides coronary vessel annotations but does not contain pixel-level stenosis annotations compatible with the ARCADE stenosis segmentation task, it was used only for the additional vessel segmentation experiments. The ICA dataset was divided into training, test, and validation sets at a ratio of 6:3:1 (368, 185, and 60 images, respectively). DCD-VMUNet and all comparison models were retrained and evaluated using the same data split and experimental protocol to ensure a fair comparison. The models trained on ARCADE were not directly used for evaluation on the ICA dataset.

All angiographic images were processed using the same preprocessing pipeline. The original images were resized to 256 × 256 pixels before being fed into the network. Image resizing was performed using bilinear interpolation, whereas segmentation masks were resized using nearest-neighbor interpolation to preserve discrete label values. Since the angiographic images are single-channel grayscale images, each image was repeated along the channel dimension to form a three-channel input. This channel replication did not introduce additional color information but was used to maintain a consistent three-channel input configuration across the evaluated network architectures. Pixel intensities were normalized before training. During training, data augmentation consisted of three operations: random horizontal flipping (*p* = 0.5), random vertical flipping (*p* = 0.5), and random rotation (*p* = 0.5), with the rotation angle randomly sampled from 0° to 360°. No additional data augmentation methods were applied.

### 2.2. DCD-VMUNet for Coronary Vessel and Stenosis Segmentation

In this study, DCD-VMUNet was proposed for pixel-level coronary vessel and stenosis segmentation in X-ray angiographic images. The vessel and stenosis segmentation tasks were intentionally trained and evaluated independently using the same network architecture. Vessel segmentation predictions were not used as priors or inputs for stenosis segmentation, thereby avoiding the propagation of vessel-segmentation errors into the lesion segmentation task and allowing the segmentation capability of DCD-VMUNet to be evaluated independently for anatomical vessel structures and pathological stenotic regions. The network was developed based on the VM-UNet architecture and further enhanced by integrating the DMSK module, centerline head, and direction head. The DMSK module was introduced to strengthen multi-scale feature extraction and improve the representation of coronary structures with diverse sizes and morphological patterns. Meanwhile, the centerline head provides auxiliary structural supervision by emphasizing the central trajectories of coronary vessels, thereby helping the model preserve vascular continuity and topological consistency. In addition, the direction head guides the network to learn directional information of elongated and tortuous vessel structures. By jointly incorporating these components, DCD-VMUNet is expected to improve the robustness and accuracy of both coronary vessel segmentation and stenosis localization. At the input stage, a 4 × 4 strided convolution is used for patch embedding. Unlike a standard convolution with a stride of 1, the convolution kernel moves across the input with a larger step size, thereby performing feature extraction and spatial downsampling simultaneously. In our implementation, this operation projects the input image into the feature space while reducing its spatial resolution from 256 × 256 to 64 × 64. Figure 2 summarizes the architecture.

#### 2.2.1. Dynamic Multi-Scale Kernel Module

The DMSK module [19] dynamically selects small-scale and large-scale convolutional kernels according to the input feature characteristics. Small-scale kernels help extract local details such as vessel boundaries and narrow branches, whereas large-scale kernels capture wider contextual information and vascular continuity. The resulting multi-scale responses are further refined by a lightweight spatial gating mechanism and residual fusion. This design allows the module to emphasize informative vessel-related regions while reducing responses from irrelevant background areas. In DCD-VMUNet, the DMSK module is applied to decoder skip connections, where it enhances feature fusion by adaptively integrating fine local details and broader contextual cues. As a result, the decoder receives more discriminative representations for coronary vessel and stenosis segmentation, while the centerline and direction heads further introduce structural and orientation-aware auxiliary supervision. The architecture of the DMSK module is illustrated in Figure 3.

To capture coronary structures at different spatial scales, the DMSK module employs two groups of candidate kernels with different receptive fields. The small-scale group consists of 3 × 3 and 5 × 5 depthwise convolutions, which focus on relatively local neighborhoods and are therefore suitable for preserving fine structures such as vessel boundaries, narrow branches, and small stenotic regions. The large-scale group consists of 7 × 7, 9 × 9, and 11 × 11 depthwise convolutions with a dilation rate of 3, providing substantially wider spatial coverage for modeling long-range vascular continuity and contextual information around bifurcations and stenotic regions. The two kernel groups are applied sequentially, allowing local structural features extracted by the selected small-scale kernel to be further enhanced using a dynamically selected large-scale receptive field.

To enlarge the receptive field without introducing the parameter cost associated with equivalently sized dense kernels, dilation is applied to the large-scale kernel group. A dilated convolution introduces spacing between adjacent kernel elements. For a kernel of size *k* and dilation rate *d*, its effective kernel size is *k*_eff_ = *k* + (*k* − 1) (*d* − 1). Accordingly, with *d* = 3, the 7 × 7, 9 × 9, and 11 × 11 kernels provide effective kernel sizes of 19 × 19, 25 × 25, and 31 × 31, respectively. All candidate convolutions are implemented as depthwise convolutions to limit the computational overhead associated with the enlarged receptive fields.

Kernel selection is performed adaptively according to the input feature representation rather than using a fixed kernel for all samples. For each kernel group, global average pooling first summarizes the input feature map into a channel descriptor, which is projected by a 1 × 1 convolution to generate a score for each candidate kernel. A Softmax operation converts these scores into normalized selection probabilities. A straight-through argmax (STE) operation is then used to obtain a hard one-hot selection during the forward pass, such that one candidate kernel from each group is activated for the current input. The STE mechanism enables this discrete kernel-selection process to remain trainable through backpropagation. Consequently, the DMSK module can dynamically select different receptive-field configurations according to the characteristics of the input features rather than relying on a predetermined kernel size. The adaptive kernel selection process is mathematically formulated as follows:

$$x_{GAP}^{l}=GAP(x_{in}^{l})$$

The selection weights for the small-scale kernels, denoted as $w_{s}$, are calculated as follows:

$$w_{s}=Softmax({Conv}_{s}^{l}(x_{GAP}^{l}))$$

where ${Conv}_{s}^{l}$ denotes a 1 × 1 convolutional layer that projects the input channel dimension onto the small-scale kernel selection space with (|L_s|) candidates. Similarly, the selection weights $w_{b}$ associated with the large-scale kernel set are derived through an independent convolutional projection:

$$w_{b}=Softmax({Conv}_{b}^{l}(x_{GAP}^{l}))$$

where ${Conv}_{b}^{l}$ denotes an independent 1 × 1 convolutional projection that maps the input channel descriptor to the large-scale kernel selection space, with its dimensionality corresponding to the number of candidate large-scale kernels (|L_b|). Accordingly, $w_{s}$ and $w_{b}$ represent the selection probability distributions over the small-scale and large-scale kernel sets, respectively, allowing the model to adaptively identify the most appropriate kernel configurations for each input sample.

After obtaining the kernel selection probabilities, the corresponding kernel indices are determined according to the learned weights. Since this operation involves discrete selection and may hinder gradient propagation, the Straight-Through Estimator (STE) [20] is adopted to maintain differentiability during training:

$${\hat{k}}_{s}={STE}_{argmax}(w_{s})$$

$${\hat{k}}_{b}={STE}_{argmax}(w_{b})$$

where ${\hat{k}}_{s}$ and ${\hat{k}}_{b}$ denote the selected kernel indices from the small-scale and large-scale kernel sets, respectively. During the forward pass, the kernel with the highest selection probability is used as the hard discrete choice for each kernel group. During backpropagation, the STE approximates the gradient through this non-differentiable selection using the underlying differentiable selection probabilities, allowing the kernel-selection layers to be optimized jointly with the rest of the network. The selected kernels are subsequently applied for multi-scale feature extraction.

After determining the optimal small-scale and large-scale kernels, the selected kernels are applied in a cascaded manner with gradually enlarged kernel sizes and dilation rates. This strategy offers two main benefits. First, contextual cues can be progressively aggregated across the receptive field, allowing the effective receptive field to expand step by step [21]. Second, features obtained from larger receptive fields provide richer contextual information, which helps the DMSK module generate more discriminative and informative representations. Specifically, the input features are first projected into a lower-dimensional feature space and then sequentially processed by the dynamically selected kernels:

$$\left\{\begin{array}{c} \;x^{l}=Project\left(x_{in}^{l}\right),\\ x_{1}^{l}={DWConv}_{{\hat{k}}_{s}}\left(x^{l}\right),\\ x_{2}^{l}={DWConv}_{{\hat{k}}_{b}}(x_{1}^{l}). \end{array}\right.$$

The concatenated multi-scale responses are subsequently reweighted through a lightweight spatial gating mechanism and integrated with residual connections to preserve the original feature representation.

#### 2.2.2. Centerline Head and Direction Head

The auxiliary structural targets were generated independently for the vessel and stenosis segmentation tasks from their corresponding task-specific ground-truth masks.

For the centerline head, a centerline-aware distance transform map (CDTM) [22] is generated from the target mask to provide structural supervision. Different from a binary centerline label, CDTM assigns the highest response to vessel centerline pixels and gradually decreases the response according to the distance from each vessel pixel to its nearest centerline point. Specifically, a one-pixel-wide centerline L is first extracted from the task-specific binary ground-truth mask using morphological skeletonization. Given a pixel $x_{i}$, the CDTM $C\left(x_{i}\right)$ is defined as:

$$C\left(x_{i}\right)=\left\{\begin{array}{c} exp\left(-\eta \times \frac{d_{x_{i}}^{2}}{\sigma_{x_{i}}^{2}}\right),\;if\;Y_{M}(x_{i})=1\\ 0,\;{if\;Y}_{M}(x_{i})=0 \end{array}\right.$$

where $Y_{M}$ denotes the ground-truth vessel mask, η = 2 is a coefficient controlling the response attenuation. The term $d_{x_{i}}$ represents the minimum Euclidean distance between pixel $x_{i}$ and the vessel centerline (L), which is calculated as:

$$d_{x_{i}}=\min\left|\left|x_{i}-x_{c}\right|\right|,x_{c}\in L$$

$\sigma_{x_{i}}$ is determined by the local radius of the centerline point nearest to $x_{i}$. The local radius of a centerline point $x_{c}$ is obtained from the Euclidean distance transform of the binary mask and is defined as its minimum distance to the background:

$$r(x_{c})=\min\left|\left|x_{c}-x_{B}\right|\right|,x_{b}\in B$$

where *B* denotes the set of background pixels. For $x_{i}$,$\sigma_{x_{i}}=r(x_{c}^{*})$ where $x_{r}^{*}$ is its nearest centerline point. The centerline head is trained to regress this CDTM, enabling the network to assign stronger attention to the central trajectories of target structures. This design is beneficial for preserving coronary artery continuity and improving the segmentation of small branches and subtle stenotic regions.

The direction head formulates local orientation estimation as a two-channel vector regression task. For each skeleton pixel, skeleton points within an 11 × 11 neighborhood (radius $r_{d}=5$ pixels) are collected, and principal component analysis (PCA) is used to estimate the local orientation. The eigenvector $v=\left(v_{x},v_{y}\right)$ corresponding to the largest eigenvalue is taken as the principal direction, and the local direction angle is calculated as:

$$\theta =atan2(v_{y},v_{x})$$

To handle the π-periodicity of the orientation, where θ and θ+π represent the same axial orientation, the direction is encoded using a double-angle representation:

$$D=\left[\cos\left(2\theta \right),sin(2\theta )\right]$$

A valid-direction mask is used to exclude unreliable orientation estimates. Branch-point candidates are identified as skeleton pixels with at least three skeleton neighbors in their 8-connected neighborhood, and their surrounding regions are excluded from direction supervision. At least three skeleton pixels within the local neighborhood are required for reliable PCA estimation; otherwise, the corresponding direction is marked as invalid. Small structures are retained for centerline supervision but are excluded from direction supervision when insufficient skeleton support is available for reliable orientation estimation.

For stenosis segmentation, the same procedure is applied to the binary stenosis masks. In this case, the centerline and direction targets represent the medial structure and local principal orientation of the annotated stenotic region, respectively, rather than the centerline of the entire coronary artery tree or the blood-flow direction.

To maintain geometric consistency during data augmentation, rotation and flipping are first applied simultaneously to the image and its ground-truth mask, after which the centerline, local radius, CDTM, direction target, and valid-direction mask are recalculated from the transformed mask. Therefore, no additional analytical transformation of the direction target is required. A masked cosine loss is applied only to valid direction pixels. If a mini-batch contains no valid direction pixels, the direction loss is set to zero to avoid division by zero and the introduction of unreliable direction supervision.

#### 2.2.3. Loss Functions

To optimize DCD-VMUNet, a multi-task loss function was employed to jointly supervise the segmentation head, centerline head, and direction head. The segmentation head is responsible for predicting the final vessel or stenosis mask, whereas the centerline and direction heads provide auxiliary structural supervision to enhance vascular continuity and directional consistency. The overall loss function is defined as:

$$L_{total}=L_{seg}+\lambda_{C}L_{centerline}+\lambda_{D}L_{Direction}$$

where $L_{seg}$, $L_{centerline}$, and $L_{Direction}$ denote the segmentation loss, centerline loss, and direction loss, respectively. In this study, the weighting coefficients $\lambda_{C}$ and $\lambda_{D}$ were set to 0.3 and 0.1, respectively.

For both the segmentation head and the centerline head, a combined binary cross-entropy and Dice loss was adopted. The segmentation loss and centerline loss are formulated as:

$$L_{seg}=L_{BCE}^{seg}+L_{Dice}^{seg}$$

$$L_{centerline}=L_{BCE}^{centerline}+L_{Dice}^{centerline}$$

Although the two losses share the same mathematical form, they supervise different prediction targets. $L_{seg}$ is used to optimize the predicted vessel or stenosis mask, while $L_{centerline}$ supervises the predicted centerline map generated from the corresponding ground-truth mask. Since the network outputs logits, a sigmoid activation function is first applied to obtain the probability map:

$$P_{i}=\delta ({\hat{Y}}_{i})$$

where ${\hat{Y}}_{i}$ is the predicted logit of pixel *i*, and $P_{i}$ the corresponding predicted probability. The BCE loss is calculated as:

$$L_{BCE}=-\frac{1}{N}\sum_{i=1}^{N}\left[Y_{i}\log\left(P_{i}\right)+(1-Y_{i})log(1-P_{i})\right]$$

and the Dice loss is defined as:

$$L_{Dice}=1-\frac{2\sum_{i=1}^{N}P_{i}Y_{i}+s}{\sum_{i=1}^{N}P_{i}+\sum_{i=1}^{N}Y_{i}+s}$$

where $Y_{i}$ denotes the ground-truth label, *N* is the total number of pixels, and *s* is a smoothing factor. The BCE term encourages accurate pixel-wise classification, while the Dice term improves the overlap between the predicted and ground-truth regions, which is particularly beneficial for thin vessels and small stenotic regions.

For the direction head, the local vascular orientation was represented as a two-channel direction vector. A masked cosine direction loss was used to constrain the predicted direction vector to be aligned with the reference direction vector only in valid direction-supervised regions. The direction loss is formulated as:

$$L_{Direction}=\frac{\sum_{i=1}^{N}M_{i}\left(1-\frac{{\hat{D}}_{i}D_{i}}{{\left||{\hat{D}}_{i}|\right|}_{2}{\left||D_{i}|\right|}_{2}+\varepsilon }\right)}{\sum_{i=1}^{N}M_{i}}$$

where ${\hat{D}}_{i}$ and $D_{i}$ represent the predicted and ground-truth direction vectors at pixel *i*, respectively. $M_{i}$ denotes the valid direction supervision mask, and ε is a small constant used for numerical stability.

#### 2.2.4. Configuration of DCD-VMUNet

As shown in Table 1, the proposed DMSK-enhanced multi-task VMUNet adopts an encoder–decoder architecture with encoder depths of [2, 2, 2, 2] and decoder depths of [2, 2, 2, 1]. The encoder progressively extracts hierarchical features through VSS blocks, which employ two-dimensional selective scanning to capture long-range spatial dependencies. During decoding, patch expansion is used to recover spatial resolution, while DMSK modules are introduced into the 768-, 384-, and 192-channel skip connections to enhance multi-scale feature fusion. By dynamically selecting convolution kernels with different receptive fields and applying spatial attention, the DMSK modules improve the representation of fine boundaries, narrow branches, and complex bifurcation structures.

The network contains three task-specific heads for segmentation, centerline prediction, and direction estimation. These complementary tasks jointly encode regional, topological, and orientation information, thereby reducing structural discontinuities and missed thin branches. The complete model contains approximately 29.57 million trainable parameters, while the lightweight decoder configuration maintains an effective balance between segmentation performance and computational efficiency.

### 2.3. Statistical Analysis

To assess the reproducibility and statistical significance of model performance, DCD-VMUNet and the corresponding strongest comparator for each segmentation task were independently trained and evaluated over five runs using different random seeds. VM-UNet was used as the comparator for vessel segmentation, whereas MALUNet was used as the comparator for stenosis segmentation. The same random seeds were used for DCD-VMUNet and the corresponding comparator to enable run-level paired comparisons and account for run-to-run variation associated with random initialization. Results are reported as mean ± standard deviation (SD). Statistical comparisons were performed using two-sided paired t-tests based on the paired results obtained from the five matched random seeds. Statistical significance was defined as *p* < 0.05.

## 3. Results

### 3.1. Experimental Setup

Experiments were carried out with the ARCADE dataset, where we trained models to achieve segmentation of vessel structures and stenosis regions within images. All experiments were conducted on a personal computer equipped with an NVIDIA RTX 4090D GPU, a 16 vCPU Intel(R) Xeon(R) Platinum 8352S CPU @ 2.20 GHz processor, and 62 GB of RAM. The implementation was carried out in Python 3.8 using the PyTorch 1.13.0 deep learning framework.

For the vessel segmentation task, training was performed with the AdamW optimizer, an initial learning rate of 1 × 10^−3^, and weight decay of 1 × 10^−2^. A cosine annealing learning rate scheduler with Tmax = 50 epochs and a minimum learning rate of 1 × 10^−5^ was employed to improve convergence. For the stenosis segmentation task, the same optimizer and weight decay were used, while the initial learning rate was set to 1 × 10^−4^. In this task, the cosine annealing scheduler was configured with Tmax = 100 epochs and a minimum learning rate of 1 × 10^−5^, aiming to improve training stability for stenosis region localization.

To ensure a fair comparison, all comparison models within each segmentation task were trained and evaluated using the same dataset split, input resolution, preprocessing and data augmentation procedures, and task-specific training and evaluation protocols. The same optimizer, learning rate, learning-rate scheduler, and other training settings were applied to all comparison models within each task. No model-specific preprocessing or post-processing was applied.

### 3.2. Evaluation Metrics

To comprehensively evaluate the segmentation performance of DCD-VMUNet, several quantitative metrics were employed, including IoU, Precision, Recall, and F1-score.

IoU (Intersection over Union) quantified the spatial overlap between the predicted segmentation region and the corresponding ground-truth target region, with higher values indicating better agreement, and was computed as:

$$IoU=\frac{TP}{TP+FP+FN}$$

Precision quantified the predictive reliability of the model by calculating the proportion of correctly identified positive pixels among all pixels classified as positive:

$$Precision=\frac{TP}{TP+FP}$$

Recall assessed the model’s sensitivity to target regions by quantifying the proportion of correctly identified positive pixels among all ground-truth positive pixels:

$$Recall=\frac{TP}{TP+FN}$$

The F1-score was computed as the harmonic mean of precision and recall, providing a comprehensive measure of segmentation performance by accounting for both the reliability of positive predictions and the ability to identify ground-truth positive regions:

$$F1\text{-}Score=2\times \frac{Precision\times Recall}{Precision+Recall}$$

TP denotes true-positive pixels, where target regions are correctly identified by the model. FP refers to false-positive pixels, in which background or non-target regions are incorrectly classified as target regions, whereas FN represents false-negative pixels, where true target regions are missed by the model. Precision measures the reliability of positive predictions by computing the proportion of correctly identified target pixels among all pixels predicted as target regions. Recall assesses the model’s sensitivity to target regions by quantifying the proportion of correctly identified target pixels among all ground-truth target pixels. The F1-score combines precision and recall using their harmonic mean, providing a balanced evaluation of segmentation performance.

In addition to the conventional pixel-wise metrics, the centerline Dice (clDice) was introduced to evaluate the structural consistency of coronary vessel segmentation [15]. Unlike conventional region-overlap metrics such as IoU and F1-score, clDice incorporates morphological skeleton information by measuring how well the predicted vessel skeleton is contained within the ground-truth mask and how well the ground-truth skeleton is covered by the predicted mask. Therefore, clDice is particularly suitable for evaluating tubular structures such as coronary vessels. A higher clDice value indicates better agreement in vessel centerline structure and improved preservation of vascular connectivity.

$$Tprec=\frac{\left|S(P)\cap G\right|}{\left|S(P)\right|}$$

$$Tsens=\frac{\left|S(G)\cap P\right|}{\left|S(G)\right|}$$

$$clDice=\frac{2Tprec\;Tsens}{Tprec+Tsens}$$

where P and G denote the predicted vessel mask and the ground-truth vessel mask, respectively, and $S\left(\cdot \right)$ denotes the skeletonization operation. Tprec measures the fraction of the predicted skeleton contained within the ground-truth mask, whereas Tsens measures the fraction of the ground-truth skeleton covered by the predicted mask.

### 3.3. Performance Evaluation on Vessel Segmentation

To quantitatively evaluate the segmentation performance of the proposed DCD-VMUNet on coronary angiographic images from the SYNTAX subset of the ARCADE dataset, we conducted comparative analyses against several state-of-the-art deep learning segmentation models widely adopted in medical imaging tasks.

The proposed DCD-VMUNet achieved the highest scores across all evaluation metrics, with an IoU of 0.6838, precision of 0.8418, recall of 0.7846, F1-score of 0.8122, and clDice of 0.7692. Compared with VM-UNet, which obtained the second-best IoU, F1-score, and clDice, DCD-VMUNet increased the IoU by 0.0213, the F1-score by 0.0152, and the clDice by 0.0148. The proposed model also surpassed YOLOv11-X, which showed competitive recall and F1-score values, by 0.0410 in IoU and 0.0296 in F1-score (Table 2). In addition to the improvements in conventional pixel-based metrics, the higher clDice score provides complementary evidence of improved centerline-level structural consistency and preservation of vascular continuity. The consistently superior results across pixel-based and centerline-aware metrics indicate that DCD-VMUNet achieves more accurate and structurally consistent vessel segmentation than the compared methods.

In addition to the quantitative comparison, Figure 4 shows representative vessel segmentation results on the SYNTAX subset. As observed, most baseline methods can roughly locate the main vessel regions, but they still suffer from discontinuous structures, boundary deviations, or missed thin branches, especially in low-contrast areas. Although MALUNet and VM-UNet achieve relatively better vessel continuity, minor omissions and inaccurate boundaries are still visible. In contrast, the proposed DCD-VMUNet generates a more complete and coherent vessel mask, preserving thin coronary vessels and branch-like structures more effectively. These qualitative observations are consistent with the quantitative results in Table 2.

### 3.4. Performance Evaluation on Stenosis Segmentation

Stenosis segmentation is generally more challenging than vessel segmentation because stenotic regions are usually small, localized, and visually subtle. In addition, the severe class imbalance between lesion pixels and background pixels increases the difficulty of accurate segmentation. Under these conditions, the recall value is particularly important, as it reflects the model’s ability to detect true stenotic regions and reduce missed lesions. The precision value further indicates whether the predicted stenosis regions are reliable and whether the model can suppress false-positive predictions.

The experimental results demonstrate that DCD-VMUNet achieves the best overall performance for stenosis segmentation among all compared models. As shown in Table 3, DCD-VMUNet obtains the highest IoU, precision, recall, and F1-score, with values of 0.4042, 0.5826, 0.5688, and 0.5757, respectively. Compared with the baseline VM-UNet, the proposed model improves IoU, precision, recall, and F1-score by 6.45, 7.98, 5.74, and 6.86 percentage points, respectively. These consistent improvements across the evaluated pixel-based metrics indicate that DCD-VMUNet effectively enhances feature representation and achieves more accurate stenosis segmentation.

Compared with other competitive methods, DCD-VMUNet also demonstrates consistent performance improvements. In particular, MALUNet achieves the second-best performance, with an IoU of 0.3893 and an F1-score of 0.5604, whereas DCD-VMUNet further improves these values by 1.49 and 1.53 percentage points, respectively. Moreover, DCD-VMUNet achieves the highest precision of 0.5826, suggesting that the proposed model can better suppress false-positive predictions and produce more reliable stenosis localization. At the same time, its recall of 0.5688 is also the highest among all models, indicating that DCD-VMUNet maintains strong sensitivity to true stenotic regions while reducing false-negative predictions. This balanced improvement in both precision and recall is particularly important for stenosis segmentation, where lesion regions are usually small, localized, and difficult to distinguish from surrounding vascular structures.

In addition to the quantitative results, Figure 5 provides representative visual comparisons of stenosis segmentation results generated by different models. As observed from the visual results, several baseline methods tend to generate fragmented masks, miss subtle stenotic regions, or introduce scattered false-positive responses. Although MALUNet and VM-UNet can capture some major stenosis-related regions, their predictions still show boundary deviations or incomplete lesion localization. In contrast, DCD-VMUNet produces cleaner and more compact segmentation masks with fewer false-positive regions and better preservation of stenotic targets. These qualitative observations are consistent with the quantitative results in Table 3.

### 3.5. Additional Validation on the Independent ICA Dataset

To further assess whether the effectiveness of the proposed method extends beyond the ARCADE dataset, additional experiments were conducted on the independent ICA dataset. All comparison models were retrained and evaluated using the same training, validation, and test split and the same experimental protocol to ensure a fair comparison. Since the ICA dataset provides pixel-level coronary vessel annotations, the additional experiments focused on the coronary vessel segmentation task.

As shown in Table 4, DCD-VMUNet achieved the best overall performance among all compared methods on the ICA dataset, with an IoU of 0.8009, precision of 0.8952, recall of 0.8838, F1-score of 0.8894, and clDice of 0.8724. Compared with the baseline VM-UNet, DCD-VMUNet improved the IoU from 0.7676 to 0.8009 and the F1-score from 0.8685 to 0.8894.

Figure 6 presents representative qualitative comparisons on the ICA dataset. DCD-VMUNet shows closer agreement with the ground-truth vessel masks and better preservation of vascular structures across different angiographic images. Together, the quantitative and qualitative results provide additional evidence that the proposed architecture remains effective on an independent dataset and that its performance improvements are not confined to the ARCADE benchmark.

### 3.6. Failure Case Analysis

Representative failure cases of DCD-VMUNet are shown in Figure 7. For vessel segmentation, the model may fail to preserve some thin distal branches or produce incomplete boundaries in challenging angiographic images. For stenosis segmentation, small or subtle stenotic regions may be missed, and occasional false-positive regions can also occur. These examples illustrate the remaining limitations of the proposed method, particularly for fine vascular structures and small stenotic regions.

### 3.7. Statistical Significance and Reproducibility Analysis

To further assess the reproducibility of the proposed method and determine whether the performance improvements over the strongest competing methods were statistically significant, DCD-VMUNet and the corresponding comparator for each segmentation task were independently trained on the ARCADE dataset using five different random seeds (42, 1, 2, 3, and 4) under identical experimental settings. VM-UNet was used as the comparator for vessel segmentation, whereas MALUNet was used as the comparator for stenosis segmentation. The same random seeds were used for DCD-VMUNet and the corresponding comparator to enable paired statistical comparisons. The results are reported as mean ± standard deviation (SD). Two-sided paired t-tests were used for statistical comparisons, with statistical significance defined as *p* < 0.05. The results of each of the five individual training runs are provided in Supplementary Tables S1–S4.

As shown in Table 5, DCD-VMUNet demonstrated stable performance across different random initializations and consistently outperformed the corresponding comparator for each segmentation task. For vessel segmentation, DCD-VMUNet achieved an IoU of 0.6842 ± 0.0060 and an F1-score of 0.8125 ± 0.0042, compared with 0.6704 ± 0.0119 and 0.8026 ± 0.0086 for VM-UNet, respectively. Statistically significant improvements were observed in IoU (*p* = 0.0202), precision (*p* = 0.0022), F1-score (*p* = 0.0209), and clDice (*p* = 0.0006), whereas the improvement in recall did not reach statistical significance (*p* = 0.1015).

For stenosis segmentation, DCD-VMUNet achieved an IoU of 0.4042 ± 0.0059 and an F1-score of 0.5757 ± 0.0060, compared with 0.3896 ± 0.0080 and 0.5607 ± 0.0083 for MALUNet, respectively. Statistically significant improvements were observed in all evaluated metrics, including IoU (*p* = 0.0002), precision (*p* = 0.0004), recall (*p* = 0.0200), and F1-score (*p* = 0.0002). These results indicate that the performance improvements of DCD-VMUNet are generally reproducible across different random initializations and are statistically significant for most vessel segmentation metrics and all evaluated stenosis segmentation metrics compared with the respective strongest competing methods.

### 3.8. Computational Complexity Analysis

To further evaluate the computational cost of the proposed model, we compared DCD-VMUNet with its baseline VM-UNet in terms of the number of trainable parameters and FLOPs. All FLOPs were calculated using an input resolution of 256 × 256 under the same computational setting. As shown in Table 6, VM-UNet contains 27.4276 M trainable parameters and requires 4.1119 GFLOPs, whereas DCD-VMUNet contains 29.5750 M parameters and requires 5.1288 GFLOPs. Compared with VM-UNet, DCD-VMUNet increases the parameter count by approximately 7.83% and the computational cost by approximately 24.73%. Meanwhile, the IoU increases from 0.6625 to 0.6838 and the F1 score increases from 0.7970 to 0.8122. These results indicate that the proposed architectural enhancements improve segmentation performance at the expense of a moderate increase in computational complexity (Table 6).

### 3.9. Ablation Study

An ablation study was conducted to evaluate the contribution of the three proposed components of DCD-VMUNet: the DMSK module, the centerline head, and the direction head. The DMSK module was designed to enhance multi-scale feature representation, whereas the centerline and direction heads provided auxiliary geometric supervision for structural continuity and orientation awareness. Several model variants were constructed, including the baseline VM-UNet, VM-UNet with only the DMSK module, VM-UNet with DMSK and the centerline head, VM-UNet with DMSK and the direction head, VM-UNet with both the centerline and direction heads, and the full DCD-VMUNet integrating all three components. All variants were evaluated under the same experimental settings within each task. The ablation results for vessel and stenosis segmentation are summarized in Table 7 and Table 8, respectively.

The ablation results demonstrate the effectiveness of the proposed components in both vessel and stenosis segmentation tasks. For vessel segmentation, the baseline VM-UNet achieved an IoU of 0.6625 and an F1-score of 0.7970. Introducing the DMSK module alone improved the IoU and F1-score to 0.6707 and 0.8029, respectively, indicating that multi-scale feature enhancement contributes to vessel representation. When the centerline head was combined with DMSK, the F1-score further increased to 0.8074. In contrast, adding the direction head to DMSK resulted in an F1-score of 0.8026, which was slightly lower than the 0.8029 obtained with DMSK alone. The combination of the centerline and direction heads also achieved competitive results, with an IoU of 0.6758 and an F1-score of 0.8065, suggesting that structural and directional supervision can effectively guide vascular feature learning. The full DCD-VMUNet achieved the best performance, with an IoU of 0.6838 and an F1-score of 0.8122, indicating that the joint integration of all three components provided the strongest overall performance.

For stenosis segmentation, the improvements brought by the proposed components were more evident. Compared with the baseline VM-UNet, which obtained an IoU of 0.3397 and an F1-score of 0.5071, the DMSK module improved the corresponding values to 0.3523 and 0.5210. Further incorporating the centerline head or direction head substantially enhanced the performance, demonstrating that structural and directional constraints are beneficial for detecting small and subtle stenotic regions. The full DCD-VMUNet achieved the highest IoU, precision, recall, and F1-score of 0.4042, 0.5826, 0.5688, and 0.5757, respectively. These results indicate that the DMSK module, centerline head, and direction head provide complementary contributions, enabling more accurate and robust segmentation of both vessel structures and stenosis regions.

## 4. Discussion

The principal finding of this study is that DCD-VMUNet provides improved coronary vessel segmentation and pixel-level stenosis localization on the ARCADE benchmark, with its effectiveness further supported by centerline-aware evaluation, repeated-run statistical analysis, and additional vessel segmentation validation on the independent ICA dataset [16,18]. For vessel segmentation, DCD-VMUNet achieved the best overall performance among the evaluated models, indicating its ability to more accurately delineate coronary anatomy in X-ray angiographic images. For stenosis segmentation, the proposed model also achieved the highest IoU, precision, recall, and F1-score among all compared methods, demonstrating its capacity to improve both the sensitivity and reliability of stenosis localization. Together with the ablation experiments, these findings indicate that dynamic multi-scale feature extraction and auxiliary geometric supervision provide complementary information for representing both vascular structures and localized stenotic regions. They further suggest that DCD-VMUNet improves segmentation performance, especially when accurate vessel delineation, small-lesion localization, and false-positive control are all important.

These findings are consistent with previous studies showing that coronary angiography segmentation is strongly influenced by network architecture and feature integration. Fully convolutional models, nested encoder–decoder networks, and ensemble approaches have improved major-vessel delineation in X-ray angiography [11,23,24]. AngioNet also demonstrated that automated segmentation can provide a basis for subsequent anatomical and diameter-based analysis [12]. Nevertheless, coronary angiograms remain difficult to segment because of low contrast, non-uniform illumination, catheter interference, surrounding anatomical structures, and ambiguous vessel boundaries [6,7].

The improvement in vessel segmentation may partly result from the interaction between the DMSK module and the centerline and direction heads. Coronary arteries vary markedly in caliber and morphology, so a single receptive-field scale may not adequately represent both proximal vessels and fine distal branches. In DCD-VMUNet, dynamic kernels were designed to combine local details with wider contextual information, thereby improving the representation of vessels with different diameters and branching patterns. In the ablation study, although DMSK alone provided a modest improvement, its combination with structural supervision produced larger gains, suggesting that multi-scale appearance features and geometric cues act synergistically. In addition, DCD-VMUNet achieved a higher clDice than the compared methods, providing complementary evidence that the improvement extends beyond pixel-wise overlap to centerline-level structural consistency.

The larger gains observed after adding structural supervision are consistent with prior work on centerline- and direction-aware vessel segmentation. Han et al. showed that auxiliary centerline and direction tasks can guide a network to learn vascular connectivity and geometric information that may be insufficiently captured by conventional pixel-wise segmentation objectives [22]. In the present study, adding the centerline head to DMSK increased the vessel F1-score from 0.8029 to 0.8074, while the complete model reached 0.8122. These findings indicate that centerline and direction supervision can both contribute to vascular continuity and boundary localization, while the full integration of appearance, centerline, and orientation information provides the strongest overall performance.

Stenosis segmentation showed more evident benefits from the proposed design. DCD-VMUNet improved over the VM-UNet baseline across all evaluated metrics, increasing IoU, precision, recall, and F1-score by 6.45, 7.98, 5.74, and 6.86 percentage points, respectively. More importantly, DCD-VMUNet achieved the highest IoU, precision, recall, and F1-score among all compared models, indicating a balanced improvement in pixel-level stenosis segmentation performance.

Compared with MALUNet, the strongest competing method for stenosis segmentation, DCD-VMUNet further improved the IoU from 0.3893 to 0.4042 and the F1-score from 0.5604 to 0.5757. The proposed model also achieved higher precision and recall, suggesting better suppression of false-positive pixels while maintaining higher pixel-level sensitivity within annotated stenotic regions. The repeated-run analysis further showed statistically significant improvements over MALUNet across all evaluated stenosis metrics, while significant improvements over VM-UNet were observed for most vessel segmentation metrics, supporting the reproducibility of the observed performance gains across different random initializations. This balanced performance is important for pixel-level stenosis localization. High precision may improve the reliability of computer-assisted lesion localization and reduce unnecessary review burden caused by false-positive predictions, whereas improved recall indicates that a larger proportion of stenosis pixels within the annotated regions are correctly identified. Previous studies have addressed coronary stenosis using vessel-diameter measurements, image-level recognition, interactive lesion detection, and video-based assessment [25,26,27,28], highlighting that stenosis identification, localization, and quantitative grading are related but distinct tasks. Accordingly, the present findings demonstrate improved pixel-level stenosis segmentation performance on ARCADE, although the moderate absolute performance indicates that further improvement is required before clinical application.

Beyond its segmentation performance, DCD-VMUNet may provide a useful foundation for more objective and reproducible analysis of coronary angiograms. Physicians’ visual assessment of stenosis severity can differ from quantitative coronary angiography and shows variation across physicians and hospitals [5]. Quantitative coronary imaging is relevant to disease detection, treatment selection, and procedural planning [4]. In this context, automatically generated vessel and stenosis masks could support subsequent centerline extraction, vessel-diameter measurement, lesion localization, and morphological quantification. Thus, pixel-level stenosis segmentation may serve as a foundational image-analysis step for subsequent quantitative coronary assessment rather than as a direct measure of stenosis severity or physiological significance. DCD-VMUNet may therefore serve as an image-analysis component within a computer-assisted coronary assessment workflow.

However, pixel-level stenosis localization should not be interpreted as a complete clinical diagnosis. Anatomical narrowing on angiography does not always reflect the physiological significance of a lesion. In the FAME trial, fractional flow reserve-guided percutaneous coronary intervention reduced the composite rate of death, myocardial infarction, and repeat revascularization compared with angiography-guided intervention in patients with multivessel coronary disease [29]. However, the present study did not evaluate correlations with quantitative coronary angiography (QCA), percentage diameter stenosis, FFR, or therapeutic decisions; therefore, the direct clinical utility of the segmentation results for lesion grading and treatment guidance remains to be validated. Accordingly, the outputs of DCD-VMUNet should be integrated with expert interpretation and other clinical or physiological information rather than used as an independent basis for treatment decisions.

Several considerations should be noted when interpreting these findings. First, although the additional evaluation on the independent ICA dataset showed that the improvement in vessel segmentation was not confined to ARCADE, further multicenter validation is still needed to assess robustness across different angiographic systems, acquisition protocols, projection angles, and image-quality conditions [30]. Second, this study focused on frame-level segmentation, whereas future work incorporating angiographic sequences or complementary projections may provide additional information for lesion assessment. Third, the present model was evaluated as a pixel-level segmentation approach rather than a complete clinical decision-making system; correlations with QCA, percentage diameter stenosis, FFR, and therapeutic decisions were not assessed. Future studies integrating segmentation outputs with these clinical measurements are needed to further establish the clinical utility of the proposed method.

## 5. Conclusions

In this study, we proposed DCD-VMUNet, a structure-aware segmentation framework for coronary angiographic image analysis. By integrating dynamic multi-scale kernel selection with centerline- and direction-aware auxiliary supervision, the proposed model was designed to enhance both appearance representation and geometric consistency for coronary vessel and stenosis segmentation. Experimental results on the ARCADE dataset demonstrated that DCD-VMUNet achieved the best overall performance among the evaluated models for coronary vessel segmentation, with improved IoU, precision, recall, F1-score, and clDice, while repeated-run statistical analysis further supported the consistency of the observed performance improvements. For stenosis segmentation, DCD-VMUNet also achieved the highest performance across all evaluation metrics, indicating improved pixel-level segmentation of small and visually subtle stenotic regions. However, the moderate absolute performance of stenosis segmentation indicates that further methodological improvement and clinical validation are required before clinical application. The ablation study further confirmed that the DMSK module, centerline head, and direction head provide complementary contributions to segmentation performance. Additional evaluation on the independent ICA dataset further supported the vessel segmentation performance of the proposed method beyond the ARCADE benchmark. These findings suggest that combining multi-scale feature extraction with geometric supervision is effective for automated coronary angiographic segmentation. Future work will focus on further multicenter validation, sequence-level angiographic analysis, and integration with quantitative coronary assessment tools to further evaluate the clinical utility of the proposed framework.

## Figures and Tables

**Figure 1:**
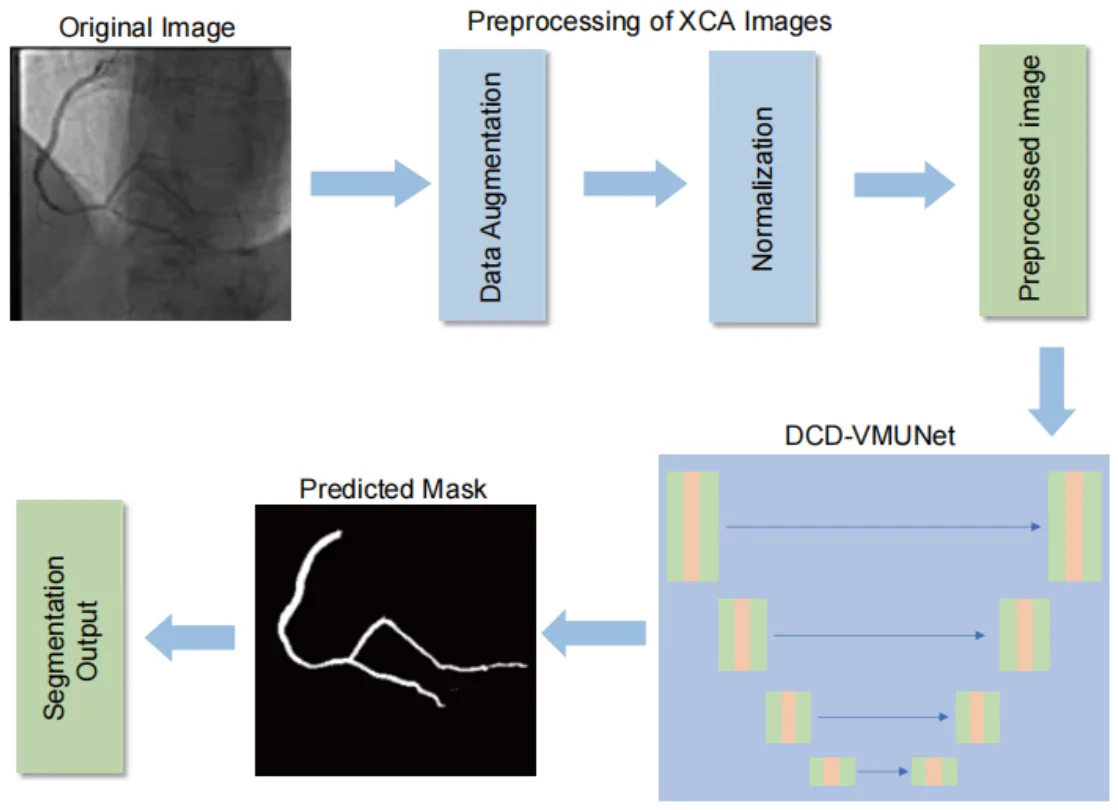
Overall workflow of the proposed DCD-VMUNet framework for coronary vessel and stenosis segmentation in X-ray coronary angiography images.

**Figure 2:**
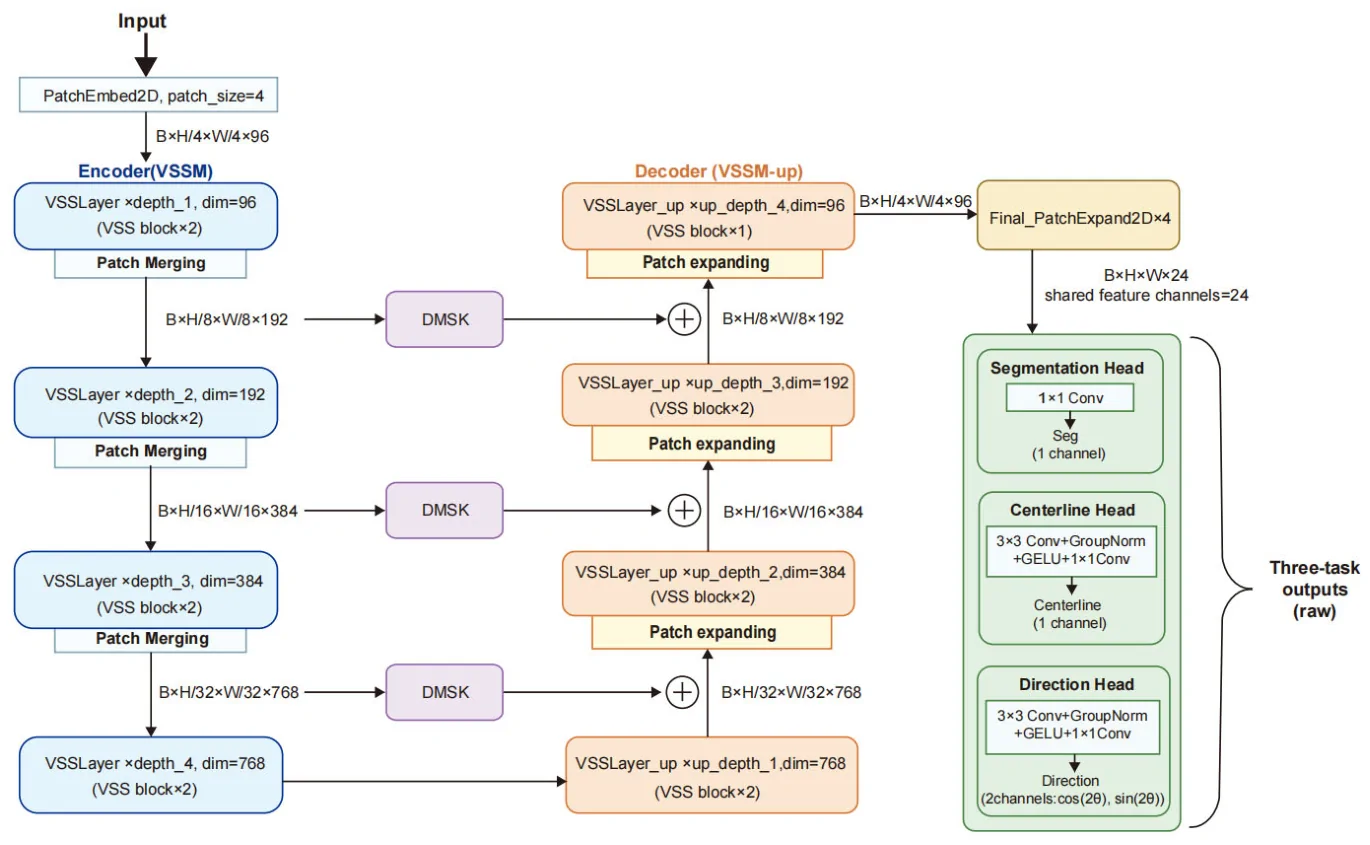
Overall network structure of DCD-VMUNet.

**Figure 3:**
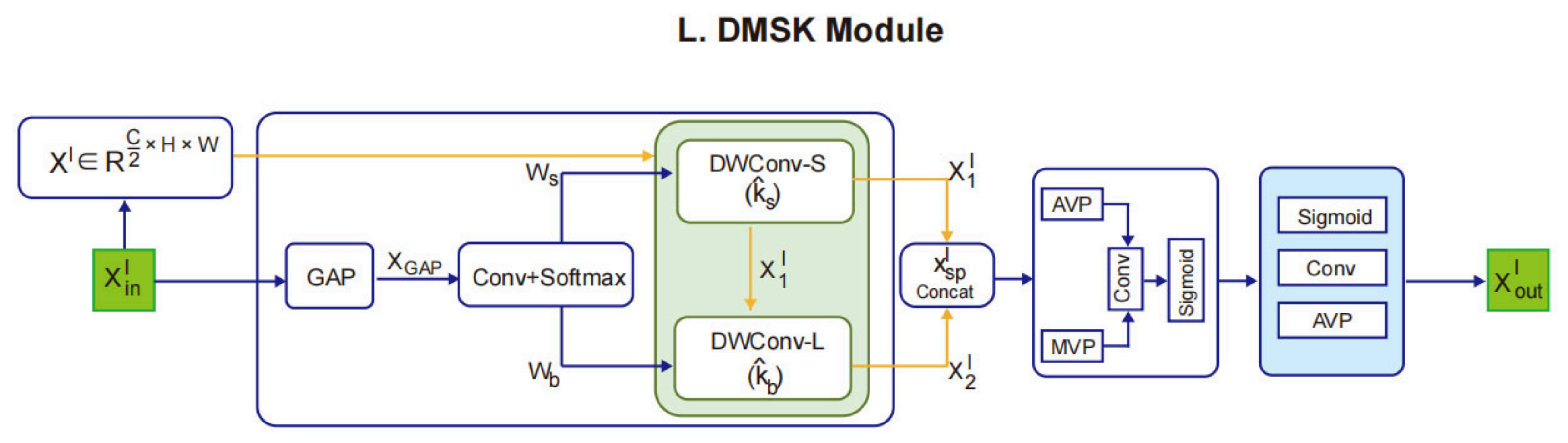
Architecture of the dynamic multi-scale kernel (DMSK) module.

**Figure 4:**
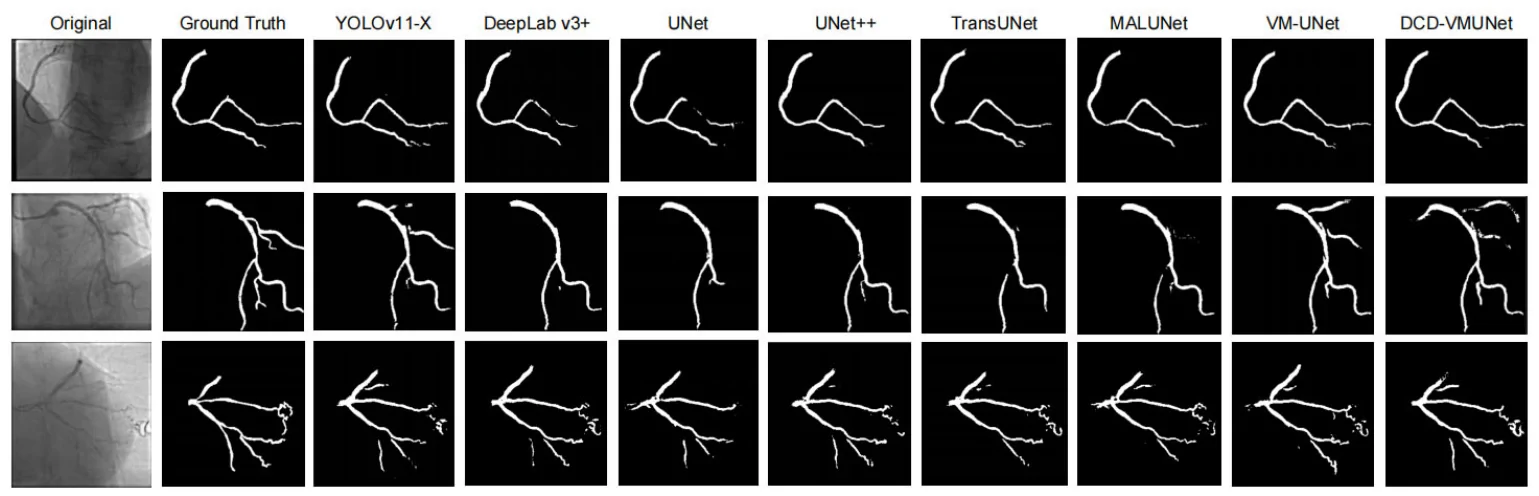
Visual comparison of vessel segmentation results on the coronary vessel segmentation subset. From left to right: original angiographic image, ground-truth vessel mask, YOLOv11-X, DeepLab v3+, UNet, UNet++, TransUNet, MALUNet, VM-UNet, and the proposed DCD-VMUNet.

**Figure 5:**
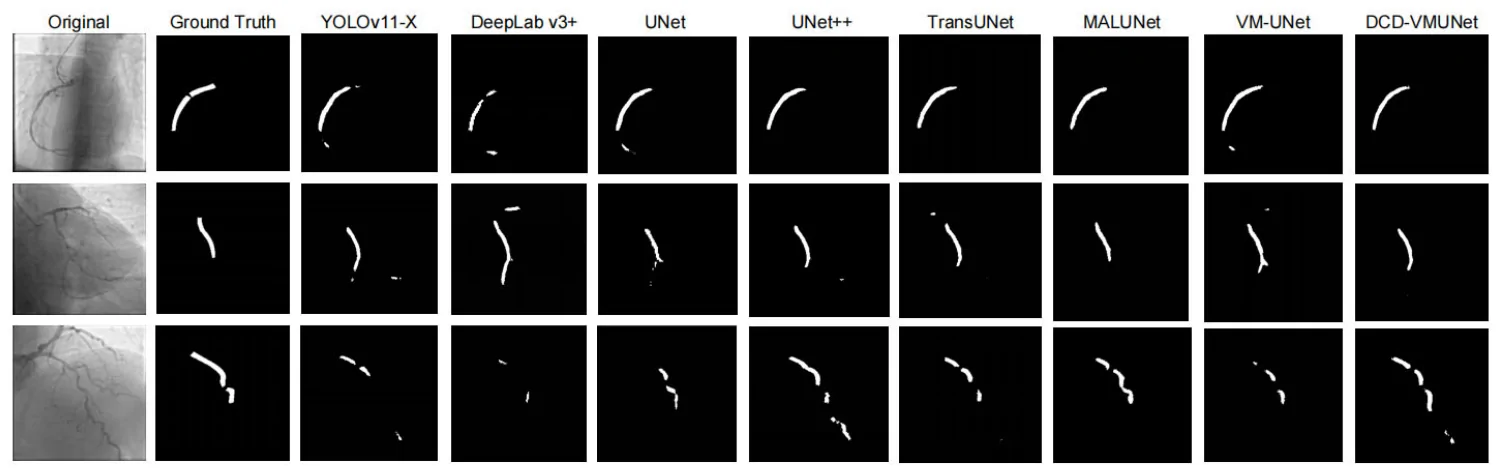
Visual comparison of stenosis segmentation results on the stenosis segmentation subset. From left to right: original angiographic image, ground-truth stenosis mask, YOLOv11-X, DeepLab v3+, UNet, UNet++, TransUNet, MALUNet, VM-UNet, and the proposed DCD-VMUNet.

**Figure 6:**
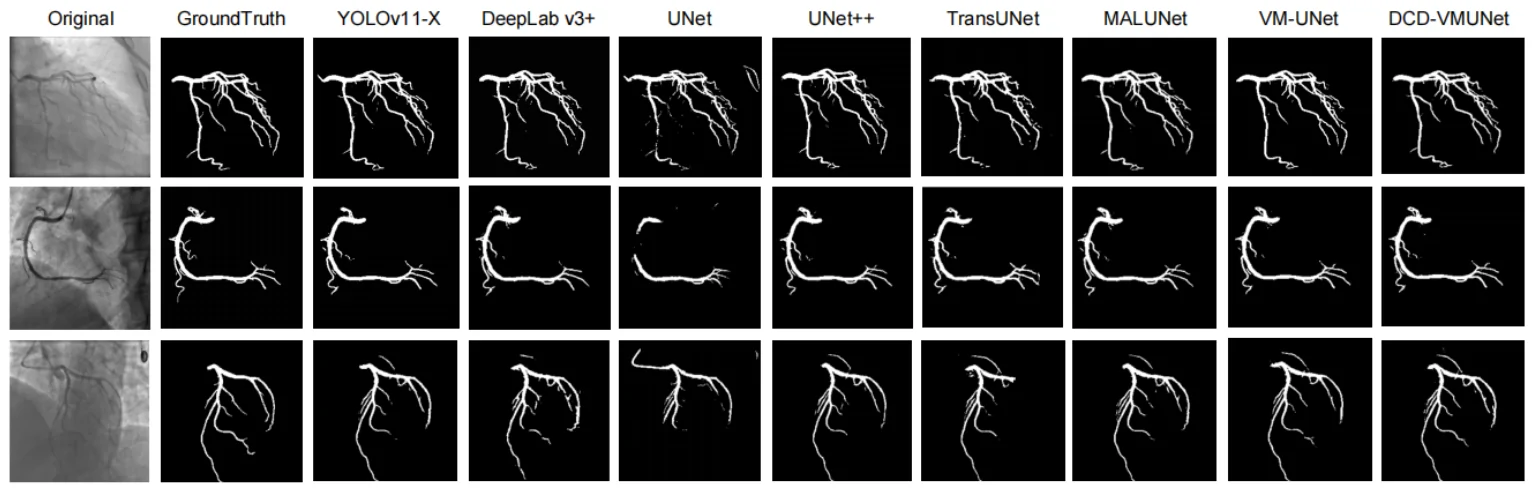
Visual comparison of coronary vessel segmentation results on the independent ICA dataset. From left to right: original angiographic image, ground-truth mask, and segmentation results obtained by YOLOv11-X, DeepLab v3+, U-Net, U-Net++, TransUNet, MALUNet, VM-UNet, and the proposed DCD-VMUNet.

**Figure 7:**
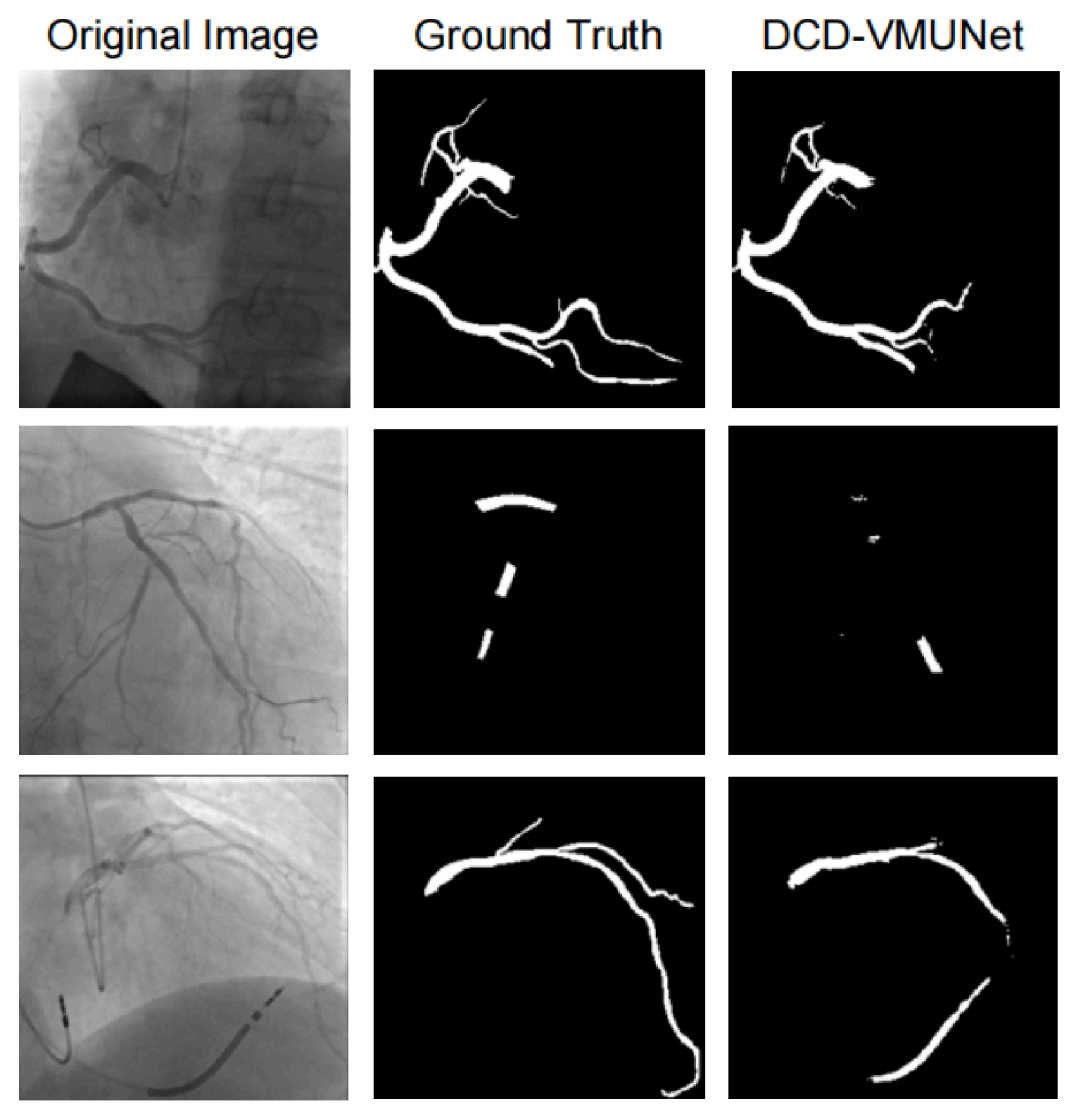
Representative failure cases of DCD-VMUNet. From left to right: original angiographic image, ground-truth mask, and DCD-VMUNet prediction.

**Table 1:** Architecture of the proposed DCD-VMUNet model.

Layer	Input Shape	Output Shape	Parameters	Features
Input	(3, 256, 256)	(3, 256, 256)	0	RGB input image; a single-channel image is repeated to three channels
Patch Embedding	(3, 256, 256)	(96, 64, 64)	4896	4 × 4 strided convolution + LayerNorm
Encoder Stage 1	(96, 64, 64)	(192, 32, 32)	285,312	2 VSS blocks + Patch Merging
Encoder Stage 2	(192, 32, 32)	(384, 16, 16)	976,128	2 VSS blocks + Patch Merging
Encoder Stage 3	(384, 16, 16)	(768, 8, 8)	3,574,272	2 VSS blocks + Patch Merging
Encoder Stage 4	(768, 8, 8)	(768, 8, 8)	8,911,872	2 VSS blocks at the bottleneck resolution
Decoder Bottleneck	(768, 8, 8)	(768, 8, 8)	8,911,872	2 VSS blocks without upsampling
Decoder Block 4	(768, 8, 8)	(384, 16, 16)	5,161,547	768-channel DMSK skip enhancement + Patch Expand + 2 VSS blocks
Decoder Block 3	(384, 16, 16)	(192, 32, 32)	1,401,227	384-channel DMSK skip enhancement + Patch Expand + 2 VSS blocks
Decoder Block 2	(192, 32, 32)	(96, 64, 64)	300,395	192-channel DMSK skip enhancement + Patch Expand + 1 VSS block
Final Expansion	(96, 64, 64)	(24, 256, 256)	36,912	4 × patch expansion + LayerNorm
Segmentation Head	(24, 256, 256)	(1, 256, 256)	25	1 × 1 convolution for binary segmentation logits
Centerline Head	(24, 256, 256)	(1, 256, 256)	5257	3 × 3 convolution + GroupNorm + GELU + 1 × 1 convolution
Direction Head	(24, 256, 256)	(2, 256, 256)	5282	3 × 3 convolution + GroupNorm + GELU + 1 × 1 convolution
Multi-task Output	Three prediction maps	Full resolution	29,574,997	Segmentation, centerline, and two-channel direction logits; total parameters

Note: Encoder depths = [2, 2, 2, 2] and decoder depths = [2, 2, 2, 1]. Shapes are reported as (C, H, W) for a 256 × 256 input. The parameter count assumes binary segmentation (num_classes = 1).

**Table 2:** Quantitative comparison of different segmentation models on the vessel segmentation task.

Model	IoU	Precision	Recall	F1-Score	clDice
YOLOv11-X	0.6428	0.7971	0.7694	0.7826	0.7453
DeepLab v3+	0.5539	0.8180	0.6607	0.7129	0.6988
UNet	0.3815	0.5736	0.5325	0.5523	0.5946
UNet++	0.5857	0.7648	0.7143	0.7387	0.7103
TransUNet	0.4173	0.6892	0.5141	0.5889	0.6357
MALUNet	0.5576	0.7246	0.7076	0.7160	0.7022
VM-UNet	0.6625	0.8349	0.7624	0.7970	0.7544
DCD-VMUNet	0.6838	0.8418	0.7846	0.8122	0.7692

**Table 3:** Quantitative comparison of different segmentation models on the stenosis segmentation task.

Model	IoU	Precision	Recall	F1-Score
YOLOv11-X	0.2775	0.5225	0.4039	0.4345
DeepLab v3+	0.2457	0.4677	0.3736	0.3945
UNet	0.2980	0.4616	0.4567	0.4591
UNet++	0.3399	0.5114	0.5033	0.5073
TransUNet	0.3582	0.5239	0.5311	0.5275
MALUNet	0.3893	0.5625	0.5583	0.5604
VM-UNet	0.3397	0.5028	0.5114	0.5071
DCD-VMUNet	0.4042	0.5826	0.5688	0.5757

**Table 4:** Quantitative comparison of coronary vessel segmentation performance on the independent ICA dataset.

Model	IoU	Precision	Recall	F1-Score	clDice
YOLOv11-X	0.7405	0.8492	0.8526	0.8509	0.8401
DeepLab v3+	0.5259	0.8031	0.6037	0.6893	0.7433
UNet	0.5103	0.8265	0.5715	0.6757	0.6007
UNet++	0.6230	0.8721	0.6858	0.7678	0.8102
TransUNet	0.5261	0.6980	0.6810	0.6894	0.6615
MALUNet	0.7019	0.7895	0.8399	0.8139	0.7892
VM-UNet	0.7676	0.8765	0.8606	0.8685	0.8639
DCD-VMUNet	0.8009	0.8952	0.8838	0.8894	0.8724

**Table 5:** Statistical comparison of DCD-VMUNet with the strongest competing methods across five independent runs with different random seeds.

Task	Metric	VM-UNet (Mean ± SD)	DCD-VMUNet (Mean ± SD)	MALUNet (Mean ± SD)	*p*-Value
Vessel	IoU	0.6704 ± 0.0119	0.6842 ± 0.0060	–	0.0202
Vessel	Precision	0.8315 ± 0.0065	0.8404 ± 0.0047	–	0.0022
Vessel	Recall	0.7757 ± 0.0152	0.7863 ± 0.0078	–	0.1015
Vessel	F1-score	0.8026 ± 0.0086	0.8125 ± 0.0042	–	0.0209
Vessel	clDice	0.7574 ± 0.0079	0.7707 ± 0.0062	–	0.0006
Stenosis	IoU	–	0.4042 ± 0.0059	0.3896 ± 0.0080	0.0002
Stenosis	Precision	–	0.5806 ± 0.0082	0.5639 ± 0.0055	0.0004
Stenosis	Recall	–	0.5708 ± 0.0042	0.5576 ± 0.0115	0.0200
Stenosis	F1-score	–	0.5757 ± 0.0060	0.5607 ± 0.0083	0.0002

VM-UNet was used as the comparator for vessel segmentation, whereas MALUNet was used as the comparator for stenosis segmentation. Data are presented as mean ± SD. *p*-Values were obtained using two-sided paired t-tests. Statistical significance was defined as *p* < 0.05.

**Table 6:** Computational complexity and segmentation performance comparison between VM-UNet and DCD-VMUNet.

	Params (M)	FLOPs (GFLOPs)	IoU	F1 Score
VM-UNet	27.4276	4.1119	0.6625	0.7970
DCD-VMUNet	29.5750	5.1288	0.6838	0.8122

**Table 7:** Ablation analysis of the proposed components in different DCD-VMUNet variants for the vessel segmentation task.

DMSK Module	Centerline Head	Direction Head	IoU	Precision	Recall	F1-Score
			0.6625	0.8349	0.7624	0.7970
√			0.6707	0.8286	0.7787	0.8029
√	√		0.6769	0.8375	0.7793	0.8074
√		√	0.6703	0.8312	0.7759	0.8026
	√	√	0.6758	0.8347	0.7802	0.8065
√	√	√	0.6838	0.8418	0.7846	0.8122

**Table 8:** Ablation analysis of the proposed components in different DCD-VMUNet variants for the stenosis segmentation task.

DMSK Module	Centerline Head	Direction Head	IoU	Precision	Recall	F1-Score
			0.3397	0.5028	0.5114	0.5071
√			0.3523	0.5172	0.5249	0.5210
√	√		0.3920	0.5663	0.5602	0.5632
√		√	0.3881	0.5586	0.5598	0.5592
	√	√	0.3921	0.5676	0.5592	0.5634
√	√	√	0.4042	0.5826	0.5688	0.5757

## Data Availability

Data available in a publicly accessible repository. The data analyzed in this study are openly available in the ARCADE dataset on Zenodo at https://doi.org/10.5281/zenodo.10390295, reference number [16].

## Supplementary Materials

The following supporting information can be downloaded at: https://www.mdpi.com/article/10.3390/bioengineering13101111/s1, Table S1. VM-UNet Vessel Segmentation; Table S2. DCD-VMUNet Vessel Segmentation; Table S3. MALUNet Stenosis Segmentation; Table S4. DCD-VMUNet Stenosis Segmentation.
