# Supplementary material for: DCD-VMUNet: A Dynamic Multi-Scale and Geometry-Aware Network for Coronary Vessel and Stenosis Segmentation in X-Ray Angiography
Source: Bioengineering (Basel). 2026 Sep 24;13(10):1111. doi: 10.3390/bioengineering13101111 (PMC13701670; doi:10.3390/bioengineering13101111)
Supplement: Supplementary file 1 [file bioengineering-13-01111-s001.zip › bioengineering-4577551-supplementary.pdf]

Table S1. VM-UNet Vessel Segmentation

Seed	IoU	Precision	Recall	F1-Score	clDice
42	0.6625	0.8349	0.7624	0.7970	0.7544
1	0.6824	0.8300	0.7932	0.8112	0.7628
2	0.6797	0.8292	0.7903	0.8093	0.7515
3	0.6540	0.8231	0.7609	0.7908	0.7498
4	0.6732	0.8404	0.7719	0.8047	0.7684

Table S2. DCD-VMUNet Vessel Segmentation

Seed	IoU	Precision	Recall	F1-Score	clDice
42	0.6838	0.8418	0.7846	0.8122	0.7692
1	0.6917	0.8366	0.7997	0.8178	0.7721
2	0.6820	0.8399	0.7839	0.8109	0.7690
3	0.6759	0.8362	0.7790	0.8066	0.7632
4	0.6876	0.8477	0.7845	0.8149	0.7802

Table S3. MALUNet Stenosis Segmentation

Seed	IoU	Precision	Recall	F1-Score
42	0.3893	0.5625	0.5583	0.5604
1	0.3844	0.5586	0.5521	0.5553
2	0.3932	0.5687	0.5604	0.5645
3	0.4010	0.5706	0.5742	0.5724
4	0.3803	0.5593	0.5430	0.5510

Table S4. DCD-VMUNet Stenosis Segmentation

Seed	IoU	Precision	Recall	F1-Score
42	0.4042	0.5826	0.5688	0.5757
1	0.4001	0.5733	0.5698	0.5715
2	0.4086	0.5861	0.5743	0.5802
3	0.4113	0.5902	0.5757	0.5829
4	0.3968	0.5710	0.5653	0.5682
